# Molecular Mechanisms Underlying Potential Pathogen Resistance in *Cannabis sativa*

**DOI:** 10.3390/plants12152764

**Published:** 2023-07-25

**Authors:** Tiziana M. Sirangelo, Richard A. Ludlow, Natasha D. Spadafora

**Affiliations:** 1 ENEA-Italian National Agency for New Technologies, Energy and Sustainable Economic Development-Division Biotechnologies and Agroindustry, 00123 Rome, Italy; 2 School of Biosciences, Cardiff University, Sir Martin Evans Building, Museum Avenue, Cardiff CF10 3AX, UK; ludlowra@cardiff.ac.uk; 3 Department of Chemical, Pharmaceutical and Agricultural Sciences, University of Ferrara, 44121 Ferrara, Italy

**Keywords:** *Cannabis*, pathogen resistance, omics, genome editing

## Abstract

*Cannabis* (*Cannabis sativa* L.) is one of the earliest cultivated crops, valued for producing a broad spectrum of compounds used in medicinal products and being a source of food and fibre. Despite the availability of its genome sequences, few studies explore the molecular mechanisms involved in pathogen defense, and the underlying biological pathways are poorly defined in places. Here, we provide an overview of *Cannabis* defence responses against common pathogens, such as *Golovinomyces* spp., *Fusarium* spp., *Botrytis cinerea* and *Pythium* spp. For each of these pathogens, after a summary of their characteristics and symptoms, we explore studies identifying genes involved in *Cannabis* resistance mechanisms. Many studies focus on the potential involvement of disease-resistance genes, while others refer to other plants however whose results may be of use for *Cannabis* research. Omics investigations allowing the identification of candidate defence genes are highlighted, and genome editing approaches to generate resistant *Cannabis* species based on CRISPR/Cas9 technology are discussed. According to the emerging results, a potential defence model including both immune and defence mechanisms in *Cannabis* plant–pathogen interactions is finally proposed. To our knowledge, this is the first review of the molecular mechanisms underlying pathogen resistance in *Cannabis*.

## 1. Introduction

*Cannabis* (*Cannabis sativa* L.) is a dicotyledonous angiosperm originating from Central Asia but is cultivated across many parts of the world due to its ability to grow in a wide range of habitats and environmental conditions [1].

*Cannabis* belongs to the Cannabaceae family and is considered one of the earliest cultivated crops, being of particular interest due to its multiple uses. Cannabinoids are responsible for the pharmacological and psychoactive properties of this crop, and these therapeutic characteristics have drawn the attention of researchers from all over the world. Additionally, hemp, a *Cannabis* variety containing less than 0.3% of tetrahydrocannabinol (THC), is cultivated for biomass and fibre, which constitute feedstock for industrial uses. Conversely, medicinal *Cannabis* contains a greater amount of THC, which has been increasing in recent years, reaching 17–28% of the dry weight in some varieties [2], or even exceeding 30% in others [3].

Among the ~130 secondary metabolites identified in *Cannabis* [4], THC, along with cannabidiol (CBD), constitute the most relevant compounds produced by this crop and are the main focus of *Cannabis* breeding programs.

Breeding efforts to produce *Cannabis* with unique fragrance and flavour characteristics are also of interest. Consequently, the profile of terpenoids, which are highly abundant and largely responsible for the characteristic aroma of *Cannabis*, is of importance, with isoprenes, monoterpenes, and sesquiterpenes being the predominant classes [5,6].

Due to the legislation regulating *Cannabis* and related breeding programs, research into the cannabinoid biosynthetic pathway is underrepresented, and it has not been sufficiently characterised, especially at the molecular level [5,7]. Many other major crops have already been widely investigated from this perspective, especially after the advent of Next Generation Sequencing (NGS) technologies [7]. However, the recent modifications in legislation and less stringent regulations [8], as well as the availability of the *Cannabis* genomic sequence [9], have broadened research in this crop, with the aim also to improve its biomass quality, in the context of sustainable agriculture [10,11].

These legislative changes have also resulted in increased *Cannabis* production and, with it, a growth of the incidence and severity of crop pathogens, along with the detection of previously unreported diseases. Among emerging pathogens of *Cannabis* recently reported there are *Botrytis cinerea* [12,13], *Fusarium* spp. [14,15], *Pythium* [16,17] *Golovinomyces* spp. [12,13], and Hop latent viroid [18], where hop (*Humulus lupulus*) is a member of Cannabaceae and is closely related to *Cannabis* [19]. These pathogens can be grouped according to the tissues they infect: root and crown (*Fusarium oxysporum*, *Fusarium proliferatum*, *Fusarium solani*, *Pythium myriotylum*, *Pythium dissotocum*, *Pythium aphanidermatum)*, leaves (*Golovinomyces* spp.), buds (Hop latent viroid) [12]. *Botrytis cinerea* is often classified as a postharvest pathogen and can attack *Cannabis* seeds, leaves, and stalks [12]. *Fusarium* and *Pythium* species are the most destructive root pathogens, especially when the infection occurs during vegetative growth. Crop losses resulting from the attack of these two pathogens can reach 30% of the total yield [12]. *Botryis* and *Fusarium* species also are harmful, as well as other fungi, such as *Golovinomyces* species, causing powdery mildew (PM, a common term for several taxa of plant pathogenic fungi), and colonizing foliar and flower tissues through the production of spores. Furthermore, extensive infection by fungi such as *Fusarium* can lead to mycotoxin accumulation in the tissues, potentially harmful to human health [20]. Hop latent viroid leads to malformation of buds and can infect other parts of the crop [18].

The above-reported fungi, oomycetes and the mentioned viroid have been investigated in *Cannabis*, as well as in several other crops, but little is known about infection within the seed, even though there are harmful pathogens, such as *Alternaria*, which can start their attack in developing seedlings [12]. The lack of significant research results on *Cannabis* bacteria pathogen defence mechanisms has also been underlined [21].

On the other hand, research into the characterization and use of biocontrol agents has consistently improved in recent years [22]. The use of synthetic fungicides to control fungal diseases has limitations due to toxicological risks, and it is necessary to replace them with safer means, for human health and with reduced environmental risks. Omics methods and their applications in the biocontrol field were recently reviewed by Massart et al. [23]. A better understanding of the molecular mechanisms underlying pathogen plant resistance can only have positive effects in this field of research.

Many of the above-reported pathogens have been identified using methods based on Polymerase Chain Reaction (PCR) of parts of rDNA, such as Internal Transcribed Spacer (ITS) and Inter Generic Spacer (IGS) regions [24]. However, for *Golovinomyces* and *Botrytis*, additional molecular markers were necessary to differentiate between species [25,26].

Few omics studies have explored the molecular mechanisms involved in *Cannabis* pathogen defence, and the underlying biological pathways need further investigation. As *Cannabis* contains many lipophilic cannabinoids and terpenoids, along with numerous other metabolites, the metabolic and genetic analyses of this crop are challenging [27]. A detailed analysis of the mechanisms involved in pathogen defence, at both the chemical and molecular level, therefore requires the use of innovative and advanced approaches.

Here, we provide an overview of *Cannabis* defence responses against some relevant pathogens, focusing on *Golovinomyces* spp., *Fusarium* spp., *Botrytis cinerea* and *Pythium*. First, we illustrate a summary of the most important molecular mechanisms of resistance, including those associated with pattern-triggered immunity in *Cannabis*, as well as the subsequent effector-triggered response activated by disease resistance genes (R genes). Secondly, for each of the previously reported pathogens, significant results of studies aimed to identify and describe genes involved in *Cannabis* defence mechanisms are reported. Many studies focus on the potential involvement of disease-resistance genes, and others draw comparisons to better-studied plants. Studies based on the use of omics science, allowing the identification of *Cannabis* candidate resistance genes, are also highlighted. They provide a starting point for genome editing approaches to generate disease-resistant crops, which are finally discussed. According to the emerging results of these studies, a potential defence model including both immune and defence mechanisms in *Cannabis* plant–pathogen interactions is proposed.

Abbreviations used throughout the manuscript are listed in Table 1.

## 2. Overview of *Cannabis* Resistance Genes to Pathogens

*Cannabis* includes genotypes whose origins are geographically very different [28], and this genetic diversity leads us to believe the existence of naturally occurring genotypes characterised by resistance to specific pathogens. Indeed, among 12 *Cannabis* genotypes evaluated, it was found that seven displayed partial or complete resistance to PM [29]. Furthermore, a recent study provided insight on the variability of *Cannabis* cultivars on disease resistance and cannabinoid accumulation over the course of crop maturation [30]. Here, PM resistance was shown for ‘FL 58’ cultivar, on which PM was never observed, as well as ‘RN13a’, ‘Otto II’, and ‘AC/DC’, cultivars, showing very low levels of PM disease.

Studies on other crop species have investigated the molecular mechanisms of resistance to *Fusarium* and PM [31,32], providing insights for further research on disease resistance responses in *Cannabis*. Conversely, the search for *Cannabis* resistance traits to viral pathogens did not yield answers so quickly [33].

Several studies focused on non-host resistance (NHR), a resistance of plant species against all non-adapted pathogens, which is considered the most durable and efficient immune system of plants, as described in the review by Oh and Choi [34]. Most non-adapted pathogen attacks are stopped by an innate defence response based on the recognition of pathogen-associated molecular patterns (PAMPs) by the plant pattern recognition receptors (PRRs), which activates PAMP-triggered immunity (PTI), also induced by reactive oxygen species (ROS) production and mitogen-activated protein kinase (MAPK) [35]. Specific PAMPs, harpin and flg22, were analyzed to study the response to *Pythium* in *Cannabis* [36]. Results showed that harpin-enhanced hemp seedlings resistant to *Pythium aphanidermatum*, while flg22 did not contribute to the defence mechanism against *P. aphanidermatum.* The lack of comprehensive experimental evidence supporting the recognition of PAMPs in *Cannabis* opens a field of future research.

The salicylic acid (SA) or the jasmonic acid (JA)/ethylene (ET) signalling pathways, which are known to have an antagonistic interaction [37], are also involved in the activation of disease resistance mechanisms. SA is involved in several key components of plant defence through complex networks, generally activated by biotrophic pathogens, and JA/ET signalling pathways are usually required for the activation of plant defence against necrotrophic pathogens [37].

However, specialised pathogens can suppress PTI responses through effector proteins, which can, in turn, activate subsequent defence responses called effector-triggered immunity (ETI) in plants with immunity to a specialised pathogen. ETI is also activated by SA or JA/ET pathway [38]. An ETI response is generally able to control specific pathogen attacks [39]. The majority of disease resistance genes in plants encode the conserved nucleotide binding site-leucine-rich repeat (NBS-LRR) disease resistance proteins [40,41], which can identify specific effectors to trigger ETI [38,42].

Studies on Wall-Associated receptor Kinases (*WAKs*) and WAK-like *(WAKLs*) genes have underlined their role in pathogen resistance across a wide range of plants [43]. The *Arabidopsis WAKL22* gene is the homolog of *Cannabis WAK7* and was shown to be responsible for dominant resistance against several *Fusarium* strains [44]. The cotton *WAK18* and *WAK29* (homolog of *CsWAK4* and *CsWAK7,* respectively), specifically expressed in flowers, showed pathogen resistance characteristics [45]. The *Juglans regia WAK9*, the homolog of *CsWAK1*, has been demonstrated to be involved in pathogen response [46]. A recent analysis of the WAK gene family in *Cannabis sativa* investigated some *CsWAKs*/*CsWAKLs* (*CsWAK1*, *CsWAK4*, *CsWAK7*, *CsWAKL1*, and *CsWAKL7*) in leaf tissues, showing how their expression differs from their homologs in other plants [47]. Furthermore, the hemp *WAK1* gene is highly expressed under drought stress conditions, and its expression can be induced by phytohormones like salicylic acid, methyl jasmonate, and ethylene [48]. These findings put the bases for future research on the potential roles of *CsWAK/CsWAKLs* in response to hormone treatments and abiotic/biotic stresses, including pathogen attacks.

Biosynthesis of specific terpenes may affect biotic and abiotic stress plant response and disease resistance [49]. For instance, few studies showed that while phytoanticipins terpenes are constitutively secreted in the absence of plant pathogen infection, phytoalexins are produced in response to pathogenic microbes [49,50]. A whole genome resequencing data across diverse samples of feral and domesticated lineages of *C. sativa*, aimed to examine their population structure, also allowed the identification of 6 loci related to stress response and 1 gene potentially involved in disease resistance [51]. This gene was annotated as mevalonate kinase (*MEV kinase*) and is involved in sesquiterpenes biosynthesis via the mevalonic acid pathway, with sesquiterpenes known for their antifungal properties [52]. Despite successful breeding efforts to modify terpene profiles, plant pathogens still constitute a significant cause of crop loss in *Cannabis* production [53].

## 3. Powdery Mildew Pathogens—*Golovinomyces* spp.

*Cannabis* is susceptible to the common PM pathogen *Golovinomyces* spp. [12]. Symptoms first appear as white circular patches of epiphytic mycelia and conidia on the leaf surface, which progress to cover the entire surface, and spread to the flowers and buds. Thaumatin-like proteins (TLPs), whose antifungal properties are thought to result from their β-1,3-glucanase activity [54], have also been associated with PM resistance in hops [55]. However, the antifungal activity of *Endochitinase* genes has not been widely studied [56]. The opposite occurs in the mildew loci O (MLO) gene family, which encodes plant proteins in conserved clades, of which clades IV and V are known for their susceptibility to PM [57]. In a recent study [58], the expression analysis of *CsMLO*s belonging to clade V, (*CsMLO1* and *CsMLO4* genes) revealed that these genes were significantly upregulated under *Golovinomyces ambrosiae* infection, confirming their possible involvement in PM susceptibility and as negative regulatory in immune system.

Subsequently, evidence was provided for the first R gene in *Cannabis*, represented by a single dominant locus able to confer complete resistance to an isolate of the PM pathogen *G. ambrosiae* [59]. Here, by using the “CBDRx” genome and linkage mapping with ~10,000 single nucleotide polymorphism (SNP) markers, 10 candidate genes of a single dominant R gene type, designed *PM1*, were detected, and this gene was mapped to a region rich in genes containing NBS and LRR domains. Specifically, a cluster of these putative disease resistance proteins contained N-terminal coiled-coil (CC) and nucleotide-binding arc (NB-ARC) domains, and two genes also contained LRR characteristics. Three annotations were also observed for tetratricopeptide repeat-containing proteins. Overall, the study identifies a key area for further research into the genetic basis for *Cannabis* resistance to *G.ambrosiae*.

NBS-LRR are involved in resistance to PM in several other species, like *Vitis vinifera* [60] and *Triticum aestivum* [61], and NBS proteins have been associated with candidate resistance genes to PM in hops [62]. According to these results and Mihalyov and Garfinkel’s [59] findings, the NBS-LRR resistance may be hypothesised for *Cannabis*.

## 4. *Fusarium* spp.

Pathogens in the genus *Fusarium* are among the most destructive in *Cannabis*, especially when affecting the roots or when infection occurs in the vegetative growth phase [10,63]. Sixteen species of *Fusarium* were reported as associated to *Cannabis* and classified into seven species complexes: *Fusarium oxysporum*, *F. solani*, *F. incarnatum-equiseti*, *F.sambucinum*, *F. tricinctum*, *F. graminearum and F. fujikuroi* [63,64]. The most evident *Fusarium* symptoms include yellowing of foliage and stem necrosis, and the related disease pathology is typically vascular wilt, but several *Fusarium* species can also result in seedling damping-off, crown rot, and reduced growth of stems and roots [12,63]. The main causative species in root and stem rots are *F. solani* and *F. oxysporum* [12].

*F. oxysporum*, a soilborne pathogen, can cause devastating vascular wilt in more than 100 plant species, and most of these fungi are formae speciales (f. sp.), an informal taxonomic group only infecting a single host plant species [65]. Two forms are reported as causal agents of *Fusarium* wilt in *Cannabis*, *F. oxysporum* f. sp. *Cannabis* (*FoxC*) and *F. oxysporum* f. sp. *vasinfectum* (*FoxV*). No host other than *Cannabis* has been reported for *FoxC* [14]. There are few studies on plant resistance to diseases caused by *Fusarium* and by *F. oxysporum* in *Cannabis* [66]. However, some resistance mechanisms and related gene families, common to a large set of plants and not specific to *Cannabis*, have been investigated [66].

The plant cell wall is the first barrier that *F. oxysporum* encounters during an attack, and this barrier defines the primary resource to fight the pathogen. Genes, which are reported to strengthen the plant cell walls, such as genes encoding 4-coumarate-CoA ligase, polyphenol oxidase and cellulose synthase, resulted upregulated in resistant Cavendish banana roots [67]. These findings suggest that the strengthened cell walls possibly confer enhanced pathogen resistance, which could also be the case in *Cannabis*.

Other gene families conferring *F. oxysporum* resistance include those involved in biosynthesis of JA and encoding P450 proteins [68]. Genes encoding dirigent-like proteins, CAP family proteins (cysteine-rich secretory proteins) and wound-responsive family proteins have also been demonstrated to be overexpressed during *F. oxysporum* infection in *Arabidopsis* [66]. NADPH (nicotinamide adenine dinucleotide phosphate) oxidases (or Respiratory burst oxidase) are also upregulated in several plants infected by *F. oxysporum*, such as wheat, cotton, and cucumber [67]. However, how these oxidases confer basal resistance to the pathogen is still unclear.

A large number of genes responsive to *F. oxysporum Arabidopsis* infection were detected in Zhu et al. [69]. Results confirmed that the ET, JA, auxin and SA pathways are all activated in response to infection by *F. oxysporum*. *WAK* gene upregulation was demonstrated to be induced upon *F. oxysporum* attack, and it seems that plants use these receptors to detect elicitors released by this fungus [69]. The induction of a number of genes encoding receptor-like kinases (RLKs) by *F. oxysporum* infection was also reported, and similar to what happens in the PM response, NBS-LRR-encoding genes also showed *F. oxysporum* resistance properties [69]. Transcription factors (TFs) play an important role as positive and negative regulators of antimicrobial compounds during the pathogenesis, and few of them belonging to the *WRKY*, *ERF*, *MYB*, and *NAC* gene families resulted constitutively up-regulated during the fungal infection [69].

Furthermore, the overexpression in *Arabidopsis* of genes belonging to the *ERF* family was related to the resistance to *F. oxysporum* [66]. Indeed, overexpression of *ERF1*, *ERF2*, and *ERF14*, which encode proteins belonging to the APETALA/ethylene (ET)-responsive-element binding protein (EREBP) family, provide resistance against this fungus. Conversely, overexpression of *ERF4* leads to decreased resistance against *F. oxysporum*.

## 5. *Botrytis cinerea*

*Botrytis cinerea* attacks over 1000 crops, including legumes, berries, and some ornamental plants [70]. Because of this wide host range, it is the most studied necrotrophic pathogen. *Cannabis* is also susceptible to the gray mold disease caused by this fungus [12,13]. This air-borne necrotrophic pathogen can attack *Cannabis* seeds, leaves, inflorescences and stalks, causing lesions covered by a conidia grey layer and often leading to broader crop decay [71]. Its pathogenic strategy consists of the secretion of enzymes to digest the plant surface, and in the synthesis of phytotoxic metabolites leading to the host cell death [72]. Generally, the infection strategies rely on several virulence factors, like toxins and plant cell wall degrading enzymes (PCWDEs), transporter proteins and enzymes that protect *B. cinerea* from oxidative stress [73].

To fight this disease, plants activate a complex network of defence pathways, which allow them to respond to the pathogen. Plant cells recognise *B. cinerea* rapidly and activate the production of pathogenesis-related proteins (PRs), and increase the production of hormones such as SA, JA, ET, abscisic acid (ABA) and brassinosteroids (BR), which have been known to play a key role in defence against this pathogen [74]. ET and JA have synergistic effects in plant *B. cinerea* resistance. JA targets the JA-Zim (JAZ) repressor for degradation, activates JA/ET-related defence genes, and SA negatively regulates this transcriptional cascade. ABA decreases resistance to *B. cinerea* through the reduction of nitric oxide (NO) formation and suppresses both ROS and ET production. BRs also regulate plant immunity mainly by interacting with TFs, playing a key role for pathway crosstalk and signal integration, and allowing regulation of plant growth and defence [74]. Findings suggest that these defence mechanisms involved in the *B. cinerea* infection process are common to several plant hosts [75].

In a recent study [76], *Cannabis* defence responses against *B. cinerea* were explored at the molecular level. Symptoms were monitored, and the expression of putative defence genes was verified in leaves by quantitative reverse transcription PCR (RT-qPCR). Five putative defence genes, involved in JA/ET-pathway (ethylene response factor 1 (*ERF1*), encoding hevein-like protein (*HEL*) and phenylalanine ammonia-lyase (*PAL*) proteins), and in SA-pathway (pathogenesis-related protein 1 (*PR1*) and pathogenesis-related protein 2 (*PR2*), were identified, showing upregulation during all infection phases and thus strongly induced by *B. cinerea* pathogen.

## 6. *Pythium*

*Pythium* sp. are classified as oomycetes and are soil-borne plant pathogens commonly referred to as water molds [77]. They cause seed death, seedling damping-off, root browning and stunting, decay of fruits and vegetables during cultivation, culminating in serious damage to a wide range of crops, like beans, opium, spinach, strawberry, soybean and tobacco [17]. *P. aphanidermatum*, typical of plants in warm regions, appeared to be the most aggressive, especially towards plants in their germination and seedling stages [17]. Together with *P. ultimum* it was reported to cause damping-off of hemp seedlings [78] and was also recently shown to cause crown and root rot on hemp [79]. Furthermore, under greenhouse hydroponic conditions, it can cause the death of mature plants [17]. Moreover, two *Pythium* species, *P. dissotocum* Drechsler and *P. myriotylum* Drechsler are able to produce zoospores at 24–27 °C, and were shown to cause root damage in *Cannabis*, resulting in browning and stunting [17]. Neither of these two species has been previously reported to infect *Cannabis* [17].

In a recent study, two PAMPs, harpin and flg22, were shown to activate immune responses in various plant species [36]. Harpin has been considered to increase *Cannabis* growth and to help its disease resistance; however, there have been no scientific studies supporting this statement. In any case, it is known that harpin induces insect defence and activates the ethylene signalling pathway [80]. Flg22 is known to induce PTI in plants, resulting in ethylene biosynthesis and activation of MAPK cascades [81].

In hemp, pretreatment with harpin was shown to enhance seedling resistance to *P. aphanidermatum* PAMPs; however, flg22 did not induce the same defence mechanism towards this pathogen species [36]. Both harpin and flg22 pretreatment induced ethylene-responsive genes, but harpin-treated seedlings showed a significant increase in *CsERF1* expression, while flg22 treatment did not affect the expression of this gene. Furthermore, both harpin and flg22-induced *CsFRK1* (FLG22-induced receptor-like kinase1) and *CsPR1*, two marker genes associated with plant innate immunity in uninfected plants

## 7. Genome Editing to Generate Disease-Resistant *Cannabis* Varieties

Omics approaches are comprehensive methods for investigating defence response pathways and have been used broadly in medicinal plants [82,83]. Furthermore, by identifying candidate resistance genes and yielding an in-depth knowledge of the underlying molecular mechanism, they provide a strong basis for genome editing studies to generate disease-resistant *Cannabis* varieties [83].

The use of genetic engineering methods in *Cannabis* to enhance its resistance to pathogens and to improve desirable traits is a subject of investigation in several research projects [84]. However, it is challenging to regenerate fully developed *Cannabis* transgenic plants [85], and, despite some candidate genes involved in pathogen resistance having been identified, functions of these genes are not yet fully validated, and only a few studies report stable transformation for *Cannabis* tissues [44].

The first edited *Cannabis* line was developed by *Agrobacterium*-mediated transformation [86], in which overexpressing the *Cannabis* developmental regulator chimera in the embryo hypocotyls of unripe grains increased the regeneration efficiency. By applying this method, the development of transgenic callus from *Cannabis* has been achieved [87]. Evidence suggests that the overexpression of Non-expressor of Pathogenesis-Related genes-1 (*NPR1*) in *Arabidopsis* can confer disease resistance to different pathogens in various plants, such as cotton [88] and *Brassica juncea* [89]. The *AtNPR1* gene has been introduced into *C. sativa* and confirmed by PCR and RT-PCR, showing that *Cannabis* can be transformed to generate disease-resistant varieties [89].

A recent mini-review on hemp genome editing [90] discusses the opportunity offered by next-generation genome editing technology. The direct delivery of CRISPR/Cas (Clustered Regularly Interspaced Short Palindromic Repeats/CRISPR-Associated Protein) ribonucleoprotein complexes into plant tissue overcomes the drawback of *Agrobacterium*-mediated transformation, by which external plasmid DNA is introduced into the crop genome. CRISPR/Cas technology, which is still less commonly used in *Cannabis*, can be applied to introduce a specific DNA fragment to a precise location in the genome. It could have broad applications in *Cannabis* breeding, modifying gene regulation and developing pathogen-resistant plants, as already performed in other recalcitrant plants, such as grapes [90]. For instance, a protocol for this type of transformation in *C. sativa* was developed, and genome-edited *Cannabis* was produced by CRISPR/Cas9 approach [90].

By using CRISPR/Cas9, the previously discussed results of the study of Mihalyov and Garfinkel [59], consisting of a set of R candidate genes, could be used as target genes to improve PM resistance in the crop.

Furthermore, results reported in other plants could provide useful inputs for *Cannabis* gene editing. For instance, the genetic transformation of wheat with TLP and glucanases resulted in enhanced resistance to *Fusarium* [91], and *MLO-7* was used as a host susceptibility (S) gene to improve grapevine and apple disease resistance to PM [92].

Overall, this advanced genome editing approach, based on a transgene-free framework, can address many problems associated with transgenic-based approaches and could be applied to produce improved non-transgenic *Cannabis*, with the most industrially desirable traits, including pathogen resistance traits.

Another alternative to *Agrobacterium* transformation protocol is represented by the use of a nanoparticle-based transient gene; through this method, multiple gene plasmids were expressed simultaneously in *Cannabis* leaf cells [93]. However, the study of disease resistance through this method is still in its infancy. It offers promising new perspectives in regulating the content of secondary metabolites, inducing pathogen resistance genes, and obtaining transgenic disease-resistant plants [94,95].

On this basis, there is a real possibility to improve *Cannabis* disease resistance by acting on targeted R genes or on S genes. A deep understanding of the underlying molecular mechanisms in which they are involved, as well as of plant-pathogen interactions, and the application of innovative molecular techniques is leading to innovations in the development of pathogen-resistant plants [96].

To date, it is still challenging to produce transgenic or gene-edited *Cannabis*, but the previously reported studies, and several gene editing approaches applied in other plant species, constitute good reference points for further *Cannabis* resistance research.

## 8. Conclusions

**Figure 1:**
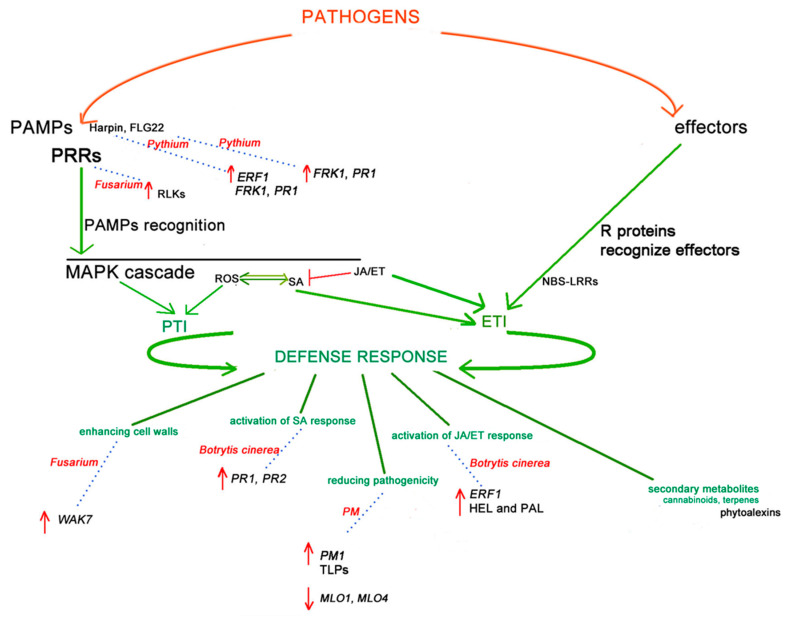
A model of *Cannabis* immune and defence responses to *PM*, *Fusarium* spp., *Botrytis cinerea* and *Pythium* interactions. The initial defence response, shared among plants, starts with two types of molecules which are derived from pathogens. Pathogen-associated molecular patterns (PAMPs), recognised by the plant pattern recognition receptors (PRRs), activate the PAMP-triggered immunity (PTI) and initiate the first plant defence response, giving a basic level of resistance to most non-specialised pathogens. Two PAMPs, Harpin and Flg22, are involved in the *Pythium* defence response, both induce *FRK1* and *PR1* and only Harpin induces *ERF1*. While PRRs, receptor-like kinases (RLKs), are involved in the *Fusarium* defence response. Reactive oxygen species (ROS) production and mitogen-activated protein kinase (MAPK) also induce PTI. Effector proteins are the other type of initiators. Effector-triggered immunity (ETI) induced by the interactions of R proteins (e.g., nucleotide binding site-leucine-rich repeat (NBS-LRR) proteins) and pathogen effectors can start the second line of host-induced defence responses. The salicylic acid (SA) and jasmonic acid (JA)/ethylene (ET) signalling pathways are involved in PTI and ETI activation and the resistance response to pathogen infections, stimulating downstream transcription factors and plant defence responses (e.g., enhancing cell walls, the activation of SA response, the reduction of pathogenicity, the activation of JA/ET response and the secondary metabolites involvement). Genes reported to strengthen the plant cell walls, such as the *WAK7* gene, are upregulated (red arrow in the model) in *Fusarium Cannabis* response. Genes involved in SA response, such as *PR1*, *PR2* (*Botrytis cinerea* response), and JA/ET response, such as *ERF1*, encoding *HEL* and *PAL* (*Botrytis cinerea* response) are all upregulated during infection. Other genes are reported to reduce virulence, such as the *PM1* gene and *MLO1* and *MLO4* (Powdery Mildew (PM) response), which are upregulated during PM infection. The phytoalexins involved in the biosynthesis of specific terpenes may also be involved in pathogen response. Arrows (in green colour) indicate positive regulation, and open blocks (in red colour) indicate negative regulation. Green lines indicate defence response mechanisms associated with different pathways. Blue pointing lines and the associated red pathogen name indicate genes/gene families involved in the defence response.

With the recent easing of legislation regulating *Cannabis* cultivation, the production and the consequent disease spread of this crop are growing rapidly. However, with this also comes a renewed interest in researching this plant from an agronomic perspective, and innovative approaches to crop improvement are being developed. Simultaneously, NGS technologies and recent advances in biotechnology have made it possible to greatly improve our understanding of *Cannabis* genetics and metabolomics, and several candidate genes involved in pathogen and resistance response have been elucidated. Figure 1 shows a potential model of immune and defence *Cannabis* responses in plant–pathogen interactions, referred in particular to PM, *Fusarium* spp., *B. cinerea* and *Pythium*, extrapolated, taking into account the studies discussed in this review. It summarises the initial plant pathogen response generally shared among plants and previously discussed in Section 2 and shows the consequent *Cannabis* defence response, detailing some genes/genes family or proteins associated with the enhancing cell walls, the activation of SA response, the activation of JA/ET response, the secondary metabolites involvement, and the overall reducing of pathogenicity, according to results discussed in Section 3, Section 4, Section 5 and Section 6.

To date, few *Cannabis* omics studies are focused on its defence mechanisms against pathogens and the associated resistance genes. However, these studies, along with omics investigations of disease resistance molecular mechanisms in other crops (see Table 2), could constitute a suitable starting point for further *Cannabis* research in this field, especially if combined with gene editing approaches which have recently made significant progress, opening new perspectives in regulating the content of secondary metabolites and inducing pathogen resistance genes.

## Figures and Tables

**Table 1:** List of abbreviations used in this manuscript.

Abbreviations	Definition
ABA	abscisic acid
BR	Brassinosteroids
CBD	Cannabidiol
CRISPR/Cas9	Clustered Regularly Interspaced Short Palindromic Repeats/CRISPR-Associated Protein
ERF	Ethylene Response Factor
ET	Ethylene
ETI	Effector-Triggered Immunity
IGS	Inter Generic Spacer
ITS	Internal Transcribed Spacer
JA	Jasmonic Acid
JAZ	JA-Zim
LRR	Leucine-Rich Repeat
MAPK	Mitogen-Activated Protein Kinase
NBS	Nucleotide Binding Site
NGS	Next Generation Sequencing
NHR	Non Host Resistance
NO	Nitric Oxide
PAL	Phenylalanine Ammonia-Lyase
PAMPs	Pathogen-Associated Molecular Patterns
PM	Powdery Mildew
PR	Pathogenesis-Related protein
PRRs	Pattern Recognition Receptors
PTI	PAMP-Triggered Immunity
PCR	Polymerase Chain Reaction
PCWDEs	Plant Cell Wall Degrading Enzymes
R genes	Resistance genes
RLK	Receptor-Like Kinases
ROS	Reactive Oxygen Species
S genes	Susceptibility genes
SA	Salicylic Acid
SNP	Single Nucleotide Polymorphism
THC	Tetrahydrocannabinol
TLP	Thaumatin-Like Protein

**Table 2:** Table summarizing the main studies examined in this review.

Pathogen	Crop	Resistance Genes/Gene Families and Proteins	References
*PM*, *Fusarium*, *Botrytis cinerea*, *Pythium*	*Cannabis*	-	[12]
PM-*Golovinomyces* spp.	*Hops*	Genes encoding NBS proteins	[62]
PM-*Golovinomyces* spp.	*Cannabis*	R gene, designated as *PM1*	[59]
PM-*Golovinomyces* spp.	*Cannabis*	Genes encoding NBS-LRR proteins	[59]
*F. oxysporum*	*Arabidopsis*	Genes encoding JA and P450 proteins	[68]
*F. oxysporum*	Resistant crops	Genes encoding 4-coumarate-CoA ligase, polyphenol oxidase, cellulose synthase	[67]
*F. oxysporum*	*Arabidopsis*	WAK gene family, genes encoding RLKs, WRKY, ERF, MYB, and NAC TFs	[69]
*F. oxysporum*	*Arabidopsis*	Genes encoding dirigent-like protein, CAP family and wound-responsive family proteins, some ERF TFs	[66]
*F. oxysporum*	*Cannabis*	*WAK7*	[47]
*Fusarium* spp.	*Cannabis*	-	[63]
*Botrytis cinerea*	Other crops	PRs, SA, JA, ET, ABA and BR gene family	[74,75]
*Botrytis cinerea*	*Cannabis*	Genes involved in JA/ET, HEL, PAL, SA, PR1 and PR2 pathways	[76]
*Pythium*	Other crops	Flg22 and PTI in plants	[81]
*Pythium*	*Cannabis*	Harpin and Flg22 PAMPs	[36]
